# Distribution of N-Acetylgalactosamine-Positive Perineuronal Nets in the Macaque Brain: Anatomy and Implications

**DOI:** 10.1155/2016/6021428

**Published:** 2016-01-03

**Authors:** Adrienne L. Mueller, Adam Davis, Samantha Sovich, Steven S. Carlson, Farrel R. Robinson

**Affiliations:** ^1^Department of Biological Structure, University of Washington, Seattle, WA 98195, USA; ^2^Department of Physiology and Biophysics, University of Washington, Seattle, WA 98195, USA

## Abstract

Perineuronal nets (PNNs) are extracellular molecules that form around neurons near the end of critical periods during development. They surround neuronal cell bodies and proximal dendrites. PNNs inhibit the formation of new connections and may concentrate around rapidly firing inhibitory interneurons. Previous work characterized the important role of perineuronal nets in plasticity in the visual system, amygdala, and spinal cord of rats. In this study, we use immunohistochemistry to survey the distribution of perineuronal nets in representative areas of the primate brain. We also document changes in PNN prevalence in these areas in animals of different ages. We found that PNNs are most prevalent in the cerebellar nuclei, surrounding >90% of the neurons there. They are much less prevalent in cerebral cortex, surrounding less than 10% of neurons in every area that we examined. The incidence of perineuronal nets around parvalbumin-positive neurons (putative fast-spiking interneurons) varies considerably between different areas in the brain. Our survey indicates that the presence of PNNs may not have a simple relationship with neural plasticity and may serve multiple functions in the central nervous system.

## 1. Introduction

Perineuronal nets (PNNs) are large accumulations of extracellular matrix molecules that form lattice-like structures around neuronal cell bodies and proximal dendrites. They consolidate around neurons near the end of developmental critical periods in V1 [1, 2] and amygdala [3]. They may restrict plasticity through a variety of mechanisms, including stabilizing synapses and inhibiting neuronal sprouting [4].

PNNs are composed of a combination of proteins and proteoglycans, which are secreted by both neurons and glia throughout early postnatal development [5, 6]. Different areas of the central nervous system have different complements of perineuronal net proteins [7]. All PNNs have four elements in common: hyaluronan, tenascin-R, link proteins, and chondroitin sulfate proteoglycans (CSPGs) [8–10]. There are four different CSPGs found in PNNs in the central nervous system: neurocan, versican, brevican, and, most frequently, aggrecan [9, 11]. Hyaluronan forms a molecular scaffold to which CSPGs adhere. These CSPG-hyaluronan connections are stabilized by link proteins. Tenascin-R then forms cross-links between these structures.

Several studies support the idea that PNNs are involved in ending critical periods of synaptic plasticity during development [2, 9, 12–14]. Critical periods in neuronal development are times during which experience can change synaptic connections. A critical period is therefore a time of activity-dependent synaptic plasticity. PNNs finish forming at approximately the same time that critical periods end and synaptic connections mature [1, 15]. PNNs grow in around neurons between postnatal days 7–14 in rat [6] and days 5–90 in rhesus macaques [16]. Artificially extending the critical period by preventing animals from acquiring experience results in a delay in perineuronal net formation [17, 18]. Dissolving PNNs in developed animals can result in at least a partial restoration of the synaptic plasticity evident during critical periods, suggesting that PNN formation is a cause, not just a correlate, of reduced plasticity [2, 3].

PNNs could inhibit synaptic plasticity either by acting as a structural barrier to formation of new processes or synapses or by inhibiting the formation of new synaptic contacts through signaling mechanisms that span the presynaptic or postsynaptic membranes. Several CSPG ligands could mediate inhibitory signals from PNNs, for example, contactin-1 [19], LAR (leukocyte common antigen-related receptor) [20], and PTP*σ* (protein tyrosine phosphatase *σ*) [21].

In addition to inhibiting plasticity, PNNs may perform other functions. They could be neuroprotective for highly active neurons that are susceptible to oxidative stress [22, 23]. For example, superoxide dismutase, an enzyme that is important for protecting against oxidative stress, binds to glycosaminoglycan side chains found abundantly on PNN CSPGs [24, 25]. PNNs may also act as cation sinks to balance the milieu around neurons with high fluctuations of ion exchanges [26]. These functions would not necessarily preclude PNN involvement in plasticity as well.

Several papers describe PNNs as primarily surrounding parvalbumin-positive inhibitory interneurons [2, 14, 27–38]. Some studies, however, also show that this correlation is inconsistent across different regions of the brain [30, 36] and even within an area [30, 39–41]. Also one study [42] showed that PNNs surround distinct subpopulations of cholinergic neurons in certain brainstem nuclei. Unfortunately, these studies compare only a limited number of areas and do not measure the proportions of neurons surrounded by PNNs in different areas.

In the cerebellum at least, PNN presence seems to be related to the amount of inhibitory input a cell receives [16]. This result complements recent data suggesting that PNNs, highly negatively charged molecules, ensure that the synapses they surround are inhibitory by influencing the relative internal and external Cl^−^ levels of the cell [43]. This proposition is very different from previous proposals which place PNNs around inhibitory interneurons and not at the targets of inhibitory interneuron input.

A reasonable start for understanding the functions of PNNs is to see where they are in the brain. To describe where PNNs occur, we surveyed PNN incidence across different regions of the primate CNS to determine where they occur and whether or not the oft-cited colocalization of PNNs and parvalbumin occurs in primates. We show which cell types are surrounded by PNNs and speculate on the possible implications of our results.

We also discuss the comparative distributions of PNNs in different CNS areas and find that PNNs are far more prevalent in the cerebellar nuclei than elsewhere in the brain. We assess the different possibilities for PNN function described above.

## 2. Materials and Methods

We collected samples of brain from nine adult (Table 1) and three developing rhesus macaques (*Macaca mulatta*), as well as one rat. All animals were sacrificed with barbiturate overdose and perfused through the left ventricle of the heart with 4% paraformaldehyde in 0.1 M phosphate buffer followed by 10%, 20%, and 30% sucrose solutions for cryoprotection.

**Figure 1:**
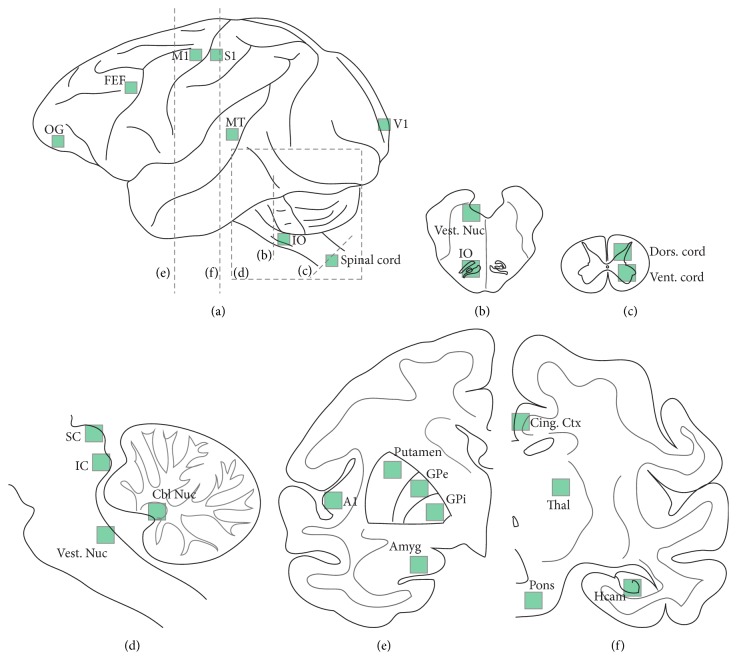
CNS of areas we examined for PNNs. (a) shows the left side of a monkey's whole brain. The green squares show the approximate size and position of our tissue samples. The dashed lines represent planes for the other subfigures. We took samples from the orbital gyrus (OG), the frontal eye field (FEF), primary motor cortex (M1), primary somatosensory cortex (S1), primary visual cortex (V1), and mediotemporal cortex (MT), the inferior olive (IO), and the cervical spinal cord. (b) shows the brainstem locations of our samples from the inferior olive (IO) and the vestibular nuclei. (c) shows the spinal cord where we sampled both the dorsal and ventral horns. (d) shows a sagittal representation of the brainstem and cerebellum where we took samples from the superior colliculus (SC), the inferior colliculus (IC), the cerebellar nuclei (Cbl Nuc), and the vestibular nuclei. (e) shows the left half of the brain in a frontal plane where we took samples from primary auditory cortex (A1), the putamen, the external globus pallidus (GPe), the internal globus pallidus (GPi), and the amygdala (Amyg). (f) shows the right half of the brain in a frontal plane where we took samples from the pontine nuclei, the hippocampus (Hcam), the thalamus (Thal), and cingulate cortex (Cing. Ctx).

After perfusion, the brain rested in a 30% phosphate-buffered sucrose solution for several days. We then took pieces from the 25 CNS locations indicated in Figure 1. From these pieces, we cut 25 *μ*m floating sections using a cryostat. Sections were stored in phosphate-buffered saline (pH 7.4). To stain for PNNs, we used* Wisteria floribunda* agglutinin conjugated to fluorescein (WFA-Flscn, 1 : 500; Vector Labs FL-1351). WFA is a lectican that binds to the long sugar side-chain components of CSPGs [37]. Although at least one study suggested that WFA is not a universal marker of PNNs [44], it has recently been shown to be an excellent marker for aggrecan (a core component in the formation of PNNs [45]) and has been routinely used as a general marker for PNNs in the past [8, 34, 36, 46–49]. WFA costains with neurocan, phosphacan, brevican, and an antiserum to nonspecified CSPGs [50]. Also in our hands, WFA and aggrecan (Cat-301 antibody, 1 : 50; Millipore MAB5284) have an extremely high degree of overlap (Figure S1 in Supplementary Material available online at http://dx.doi.org/10.1155/2016/6021428). We therefore use WFA as our proxy for PNNs for the purpose of illustrating the broad distribution of PNNs in the macaque central nervous system. We used either NeuN (mouse monoclonal neuronal nuclei N, 1 : 500; Millipore Corp., MAB377) or avidin conjugated with Texas Red (Avidin-TxRd, 1 : 500; Invitrogen, A-820) as a neuronal stain. We used NeuN to label all brain areas except the cerebellar nuclei and avidin to label cerebellar nuclear neurons [51], which are not antigenic for the NeuN antibody [52]. We also stained for a subset of GABAergic inhibitory interneurons with an anti-parvalbumin antibody (mouse monoclonal, 1 : 500, Sigma, P3088). We exposed the sections to the primary antibodies on consecutive days to maximize signal. In the case of NeuN and parvalbumin, sections were additionally exposed to the secondary antibody Alexa Fluor 568 (1 : 1000; Invitrogen, A-21124). A limited number of sections were mounted directly on slides after cryostat sectioning, stored at −80°C, and later stained with the same protocol as the floating sections [53]. These sections were stained with primary antibodies to synaptic vesicle protein 2 (rabbit polyclonal SV2, 1 : 50, Courtesy of Carlson Lab), choline acetyltransferase (ChAT, rabbit polyclonal, 1 : 500; Millipore, AB143), and/or protein tyrosine phosphatase *σ* (PTP*σ*, mouse monoclonal, 1 : 100; Millipore, MABN605). In the case of triple-stained sections, we used an Alexa Fluor 647 secondary antibody (1 : 1000; Invitrogen, A-31573). Sections were mounted with Fluoromount media and imaged on a Zeiss Axioskop 2 confocal microscope using LSM 5 Pascal software. The same excitation and acquisition parameters were used for all sections from a single staining session. We occasionally manipulated the gain and offset values of the images following collection for better cell discrimination for counting; however, we did not postprocess any of the images in this paper.

**Figure 2:**
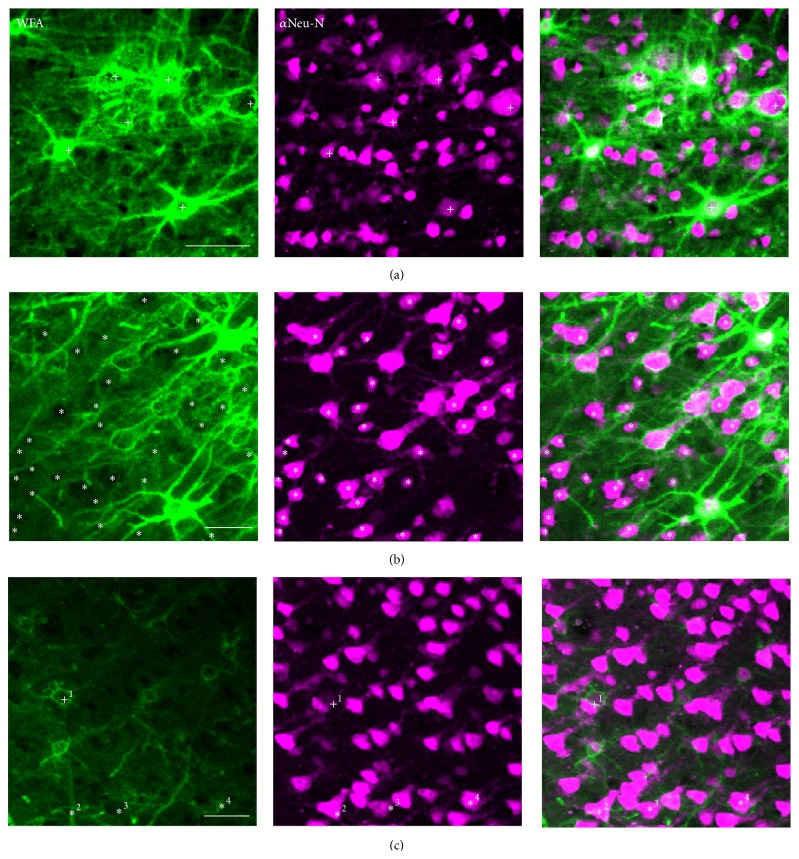
Perineuronal net identification. WFA, NeuN, and combined labeling in primary motor cortex. (a) shows labeling that we identified as PNN-positive, marked with +. (b) shows labeling that we identified as PNN-negative. Cells without PNNs are indicated with *∗*. (c) shows some examples of how we classified more ambiguous labeling. 1: a cell with strong WFA and weak NeuN staining, which we classified as WFA+ (+ symbol). 2: labeling which looks like it might surround a cell but does not clearly surround NeuN labeling (*∗* symbol). 3: cells that produce absence of background labeling in the WFA channel but are not associated with clear WFA labeling. Even though the background is brighter than the “black” region, we do not identify these cells as WFA+, because they do not have a clear ring-like net (*∗* symbol). 4: cell with partial somatic WFA staining, but because the ring around the cell is incomplete, we cannot unambiguously classify the cell as PNN-positive (*∗* symbol). Scale bars are 50 *μ*m.

We calculated the fraction of neurons surrounded by PNNs in each area by counting the number of cells with WFA staining (marking PNNs) and dividing it by the number of cells stained with NeuN or avidin (marking neurons). We identified neurons as having PNNs if the staining surrounding the cell was clearly distinguishable from the background, surrounded more than three quarters of the soma, and colocalized with neuronal staining (Figure 2). Four individuals separately counted neurons and PNNs on each image. We averaged these counts and then used these averages to calculate an average percentage of neurons surrounded by PNNs across monkeys. We used a similar procedure to count parvalbumin-positive and WFA-positive neurons but did not calculate percentages from the counts. For this study, we do not use a full stereological approach but instead describe our results in terms of proportions of neurons surrounded by nets because it facilitates comparisons between areas.

We collected tissue from eight adult male rhesus macaques (ages 5, 5, 6, 7, 8, 9, 11, and 21 years) and one adult female (age 12 years), as well as one adult rat (for comparison). The tissue from these animals was primarily assigned to other projects; therefore, we were not able to collect samples from every area in every animal. In the monkeys, we acquired samples from a minimum of four different animals for each area for the PNN/neuron comparisons. Also, in order to examine the expression of PNNs around cerebellar nuclear neurons during development, we examined tissue from one animal each at fetal day 145, postnatal day 5, and postnatal day 90. The value for each area for each animal was calculated based on an average of between 1 and 3 image frames per section and an average of 1–3 sections per area.

## 3. Results

### 3.1. Proportions of Neurons Surrounded by PNNs in Different Areas of the Brain

**Figure 3:**
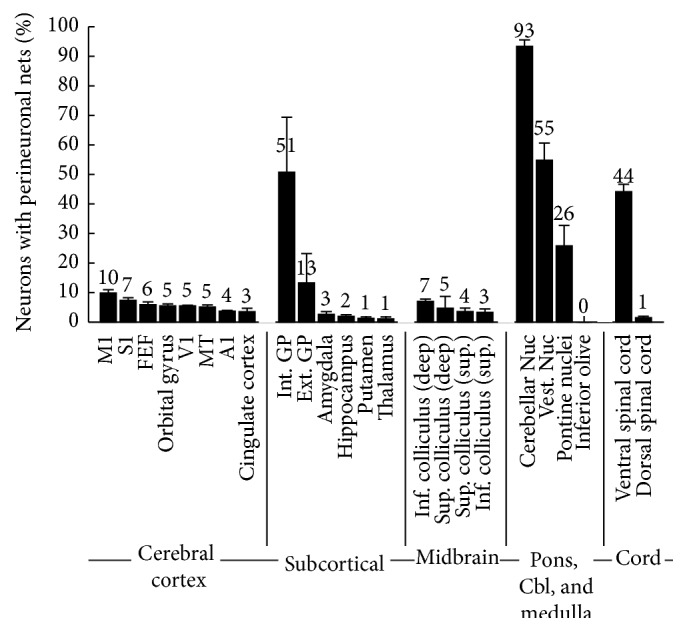
Percentage of neurons surrounded by perineuronal nets in different parts of the brain. Each bar represents the mean percentage of neurons that were surrounded by perineuronal nets in one part of the brain. The values, rounded to the nearest percentage, are shown above each bar. Averages and standard error are calculated from at least 4 monkeys for each area.

Figure 1 shows the different areas that we examined.  Figure 3 shows the percentage of neurons surrounded by PNNs in each of these areas.

#### 3.1.1. Cortex

**Figure 4:**
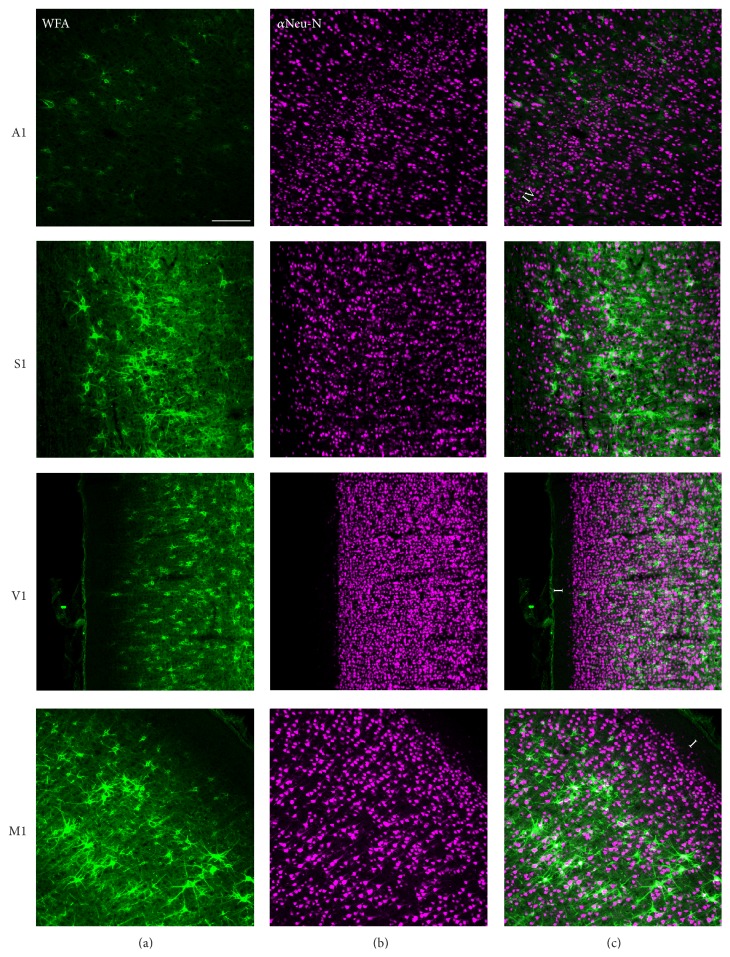
Perineuronal nets in primary sensory and motor cortex. (a) shows perineuronal nets stained with WFA. (b) shows NeuN-labeled neurons. (c) shows merged images of the two. Neurons are predominantly absent from layer I, which is primarily composed of fibers running parallel to the cortical surface. We identify layer I or layer IV, where appropriate, in the rightmost panels. A1: primary auditory cortex, S1: primary somatosensory cortex, V1: primary visual cortex, and M1: primary motor cortex. Scale bar = 200 *μ*m.

**Figure 5:**
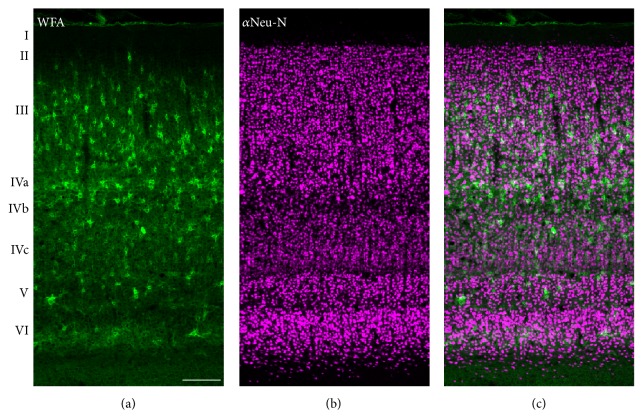
Perineuronal nets in primary visual cortex layers. (a) shows perineuronal nets stained with WFA. (b) shows NeuN-labeled neurons. (c) shows merged images of the two. Scale bar = 200 *μ*m.

Figure 4 shows examples of labeling of PNNs and neurons in primary sensory cortices (A1, S1, and V1) and primary motor cortex (M1). In these and all other areas of cerebral cortex, PNNs surround less than 10% of neurons. In all areas of the cortex that we examined, the nets surrounded neurons more frequently in layers three and four than in other layers.  Figure 5 demonstrates this pattern in V1. In addition, in M1, the large projection neurons (Betz Cells) in layer five were often surrounded by PNNs.

We also examined cingulate cortex, cortex of the orbital gyrus, area MT, and the frontal eye field (Figure S2). In these areas, approximately 5% of neurons costained with the perineuronal net marker. Of all the areas of cerebral cortex we examined, PNNs were the most abundant in primary motor cortex.

#### 3.1.2. Subcortical Areas

PNNs surrounded very few cells in the amygdala, hippocampus, and thalamus (Figure S3). The distribution of PNNs in the basal ganglia varied depending on the subregion. There were PNNs around very few neurons in the putamen (1%), more neurons in the external globus pallidus (13%), and about half of the neurons in the internal globus pallidus (51%) (Figure S4). The quality of the labeling differed between external and internal globus pallidus. WFA labeling was strong and sharply defined cells in the internal globus pallidus, but, in the external globus pallidus, the stain was weaker and more diffuse.

#### 3.1.3. Brainstem and Cerebellum

**Figure 6:**
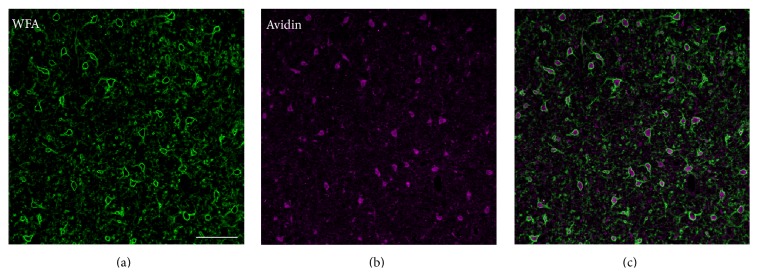
Perineuronal nets in the cerebellar nuclei. (a) shows perineuronal nets stained with WFA. (b) shows avidin-labeled neurons. (c) shows merged images of the two. Scale bar = 200 *μ*m. Note the high proportion of avidin-labeled neurons (pink) surrounded by PNNs (green).

PNNs surround an average of 93% of cells in the cerebellar nuclei, the highest percentage of any area we examined.  Figure 6 shows an example of labeling here. The area with the second densest population of PNN-labeled neurons was the vestibular nuclei (55%). The vestibular nuclei are similar to the cerebellar nuclei in that they receive direct input from inhibitory Purkinje cells in the cerebellar cortex. Our estimate of perineuronal net density combines both the medial and lateral vestibular nuclei. Qualitatively, we observed more neurons with PNNs in the lateral vestibular nucleus.

PNNs surround 26% of neurons in the pontine nuclei. In addition to well-defined labeling of a subset of neurons, WFA also diffusely stained the area between cells. Because this diffuse staining made it harder to identify PNN ensheathment, it is possible that our calculation of the proportion of cells surrounded by PNNs is an underestimate. This diffuse labeling was also noticeable in other areas of the brain as well, that is, in the ventral horn of the spinal cord and the internal globus pallidus. Neurons in the inferior olive were completely free from perineuronal net labeling (Figure S5). In both the superior and inferior colliculi, PNNs surrounded a higher fraction of deeper neurons than of superficial neurons (Figure S6).

#### 3.1.4. Spinal Cord

**Figure 7:**
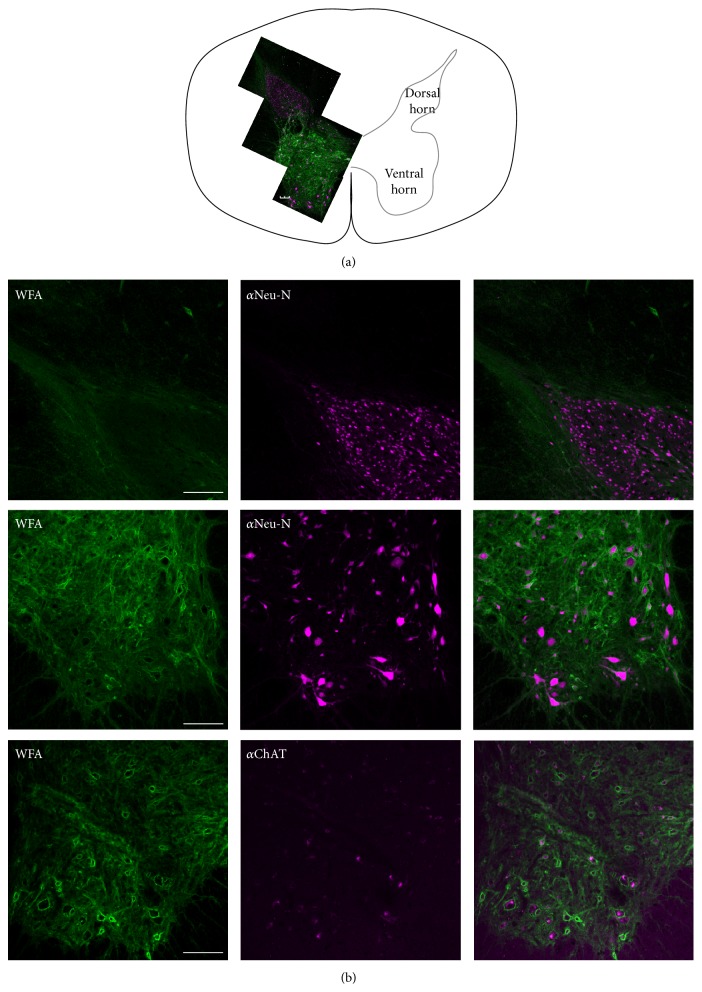
(a) Perineuronal nets in ventral and dorsal horns of the spinal cord. A composite image of perineuronal nets (stained with WFA) and neurons (stained with *α*-NeuN) is superimposed on the left of a schematic of the cervical spinal cord. (b) Perineuronal nets (WFA+, green) are more prevalent around neurons (NeuN, pink) in the ventral spinal cord (middle panels) than in the dorsal spinal cord (top panels). Cells surrounded by PNNs are often ChAT+, putative primary motoneurons (bottom panels). Scale bars = 200 *μ*m.

PNNs surround almost 50% of neurons in the ventral horn of the cervical spinal cord but almost none of the neurons in the dorsal horn.  Figure 7(a) shows a composite picture of labeling in the dorsal and ventral horns of the spinal cord that illustrates the large difference in the frequency of WFA+ and WFA− cells. Of the WFA+ neurons in the ventral horn, 90% (±3.2%) costained with a marker for primary motoneurons, an anti-choline acetyltransferase (ChAT) antibody. 75.6% (±1.8%) of ChAT-positive cells were surrounded by WFA+ PNNs (Figure 7(b)).

### 3.2. PNN Presence around Parvalbumin-Positive Inhibitory Interneurons Varies between Areas

**Figure 8:**
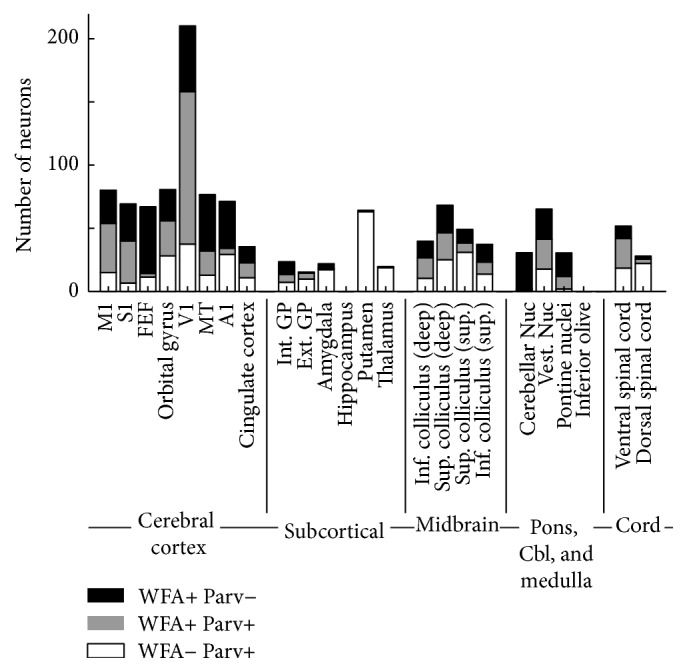
Relative frequencies of perineuronal net- and parvalbumin-positive cells in different areas of the brain of one male animal, age 5. Cells were counted in 0.92 mm^2^ areas for each region listed. We tallied cells that stained positive for either PNNs (WFA+ Parv−, black) or parvalbumin alone (WFA− Parv+, white) as well as cells that stained for both markers (WFA+ Parv+, gray) separately. The regions examined were, from left to right, primary motor cortex (M1), primary somatosensory cortex (S1), frontal eye field (FEF), orbital gyrus, primary visual cortex (V1), mediotemporal cortex (MT), primary auditory cortex (A1), cingulate cortex, internal globus pallidus (int. GP), external globus pallidus (ext. GP), amygdala, hippocampus, putamen, thalamus, deep inferior colliculus, deep superior colliculus, superficial superior colliculus, superficial inferior colliculus, cerebellar nuclei (Nuc), vestibular nuclei (Vest. Nuc), pontine nuclei, inferior olive (IO), ventral (V) spinal cord, and dorsal (D) spinal cord.

We counted the number of parvalbumin-positive cells in a given (0.94 mm^2^) area for each of the regions shown in Figure 1 that either did or did not costain for PNNs. As Figure 8 summarizes, we found that (1) not all parvalbumin-positive neurons are surrounded by PNNs; (2) not all neurons with PNNs are parvalbumin-positive; (3) the relative frequencies of these two types of cells (WFA+ and parvalbumin+) differed widely between areas. For example, in primary visual cortex, the populations of PNN-positive and parvalbumin-positive cells were mostly overlapping. However, in the frontal eye field, the two populations were almost completely distinct (Figure S7).

In noncortical parts of the brain, regions in which PNNs are scarce do not exhibit corresponding lack of parvalbumin-positive cells. For example, Purkinje cells in the cerebellar cortex stain strongly for parvalbumin, but only weakly for PNNs (Figure S8). The same is true for neurons in the dorsal horn of the spinal cord (Figure S9). Also, excitatory projection neurons in the cerebellar nucleus, which are densely surrounded by PNNs, are not parvalbumin-positive.

These results show that although in some areas of the brain PNNs surround many parvalbumin-positive inhibitory neurons, this is not a general phenomenon throughout the brain.

### 3.3. WFA+ Neurons in the Cerebellar Nuclei Do Not Express PTP*σ* at the Cell Surface

**Figure 9:**
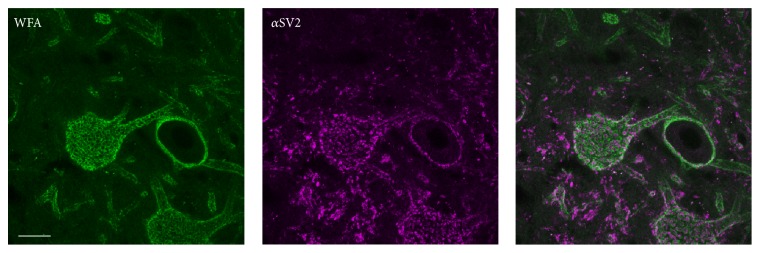
Perineuronal nets surround synapses in cerebellar nuclear neurons. WFA-positive PNNs (green) ensheath the soma and proximal dendrites of neurons in the cerebellar nucleus (pink). They form openings around synapses, labeled with an antibody to synaptic marker SV2 (pink). *Z*-projection of 10 um confocal stack. Scale bar = 20 *μ*m.

**Figure 10:**
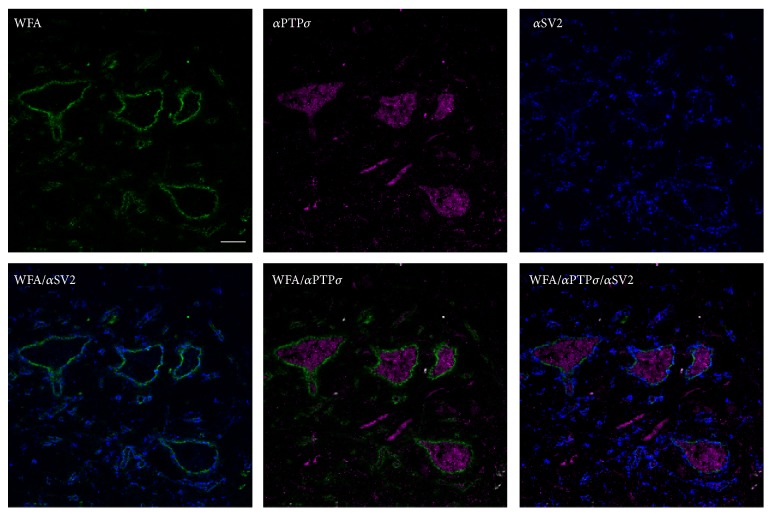
PTP*σ* does not colocalize with PNNs or synapses. Expression of PNNs (stained with WFA, green), PTP*σ* (pink), and synapses (stained with anti-SV2 antibody, blue) do not overlap. Scale bar = 40 *μ*m.

We examined whether PNNs interfaced with PTP*σ*. Such an interface might allow PNNs to inhibit synaptic connections through intracellular signaling events, a function in which PTP*σ* is implicated [54, 55]. If this were the case, we would expect to see PTP*σ* colocalizing with WFA, possibly at positions proximal to synapses, so as to inhibit expansion of synaptic sites. Further, we would expect to find PTP*σ* expressed on cells presynaptic to the neurons heavily surrounded by PNNs.  Figure 9 shows that PNNs in the cerebellar nuclei surround the cell bodies and dendrites of large neurons, leaving gaps for synaptic contacts. We examined these neurons and found that 75.5% of cerebellar nuclear neurons exhibit cytosolic PTP*σ*. Based on their punctate expression pattern, it is possible that PTP*σ* is localized to endosomes. 90% of all PTP*σ*+ neurons were also WFA+. Nonetheless, the expression of PTP*σ* was primarily cytosolic and did not appear to be linked to the location of synapses (Figure 10).

### 3.4. PNN Prevalence in Different Age Animals

**Figure 11:**
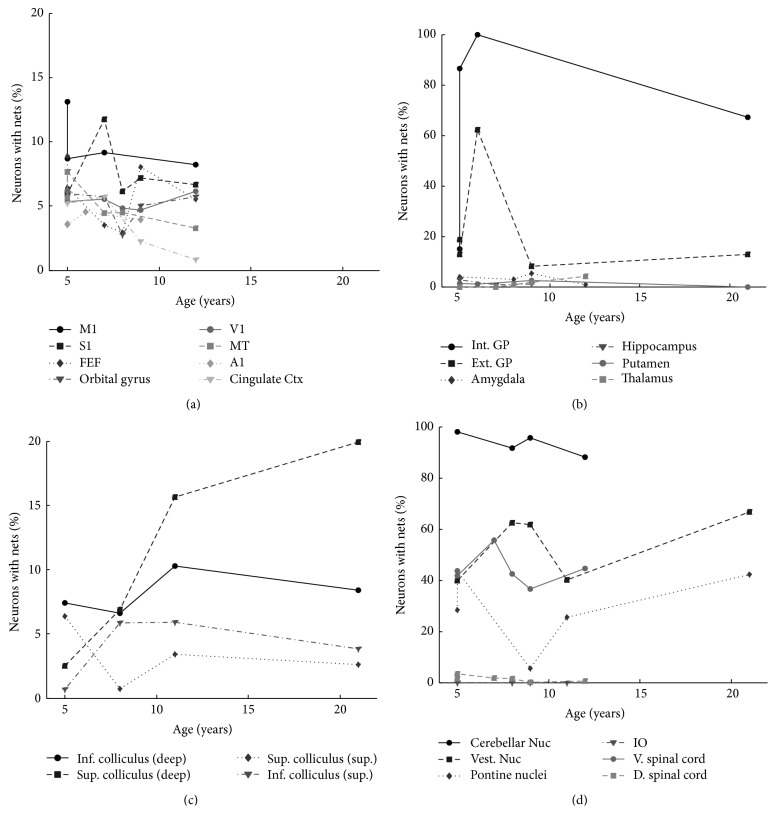
Percentage of neurons with PNNs across animals of different ages. Percentage of neurons surrounded by PNNs in (a) the cerebral cortex, (b) subcortical structures, (c) the colliculi, and (d) the cerebellar nuclei, brainstem, and spinal cord. Each symbol represents a different area in the subplot (see legends).

PNNs appear during early postnatal development but we know little about how the prevalence of neurons surrounded by PNNs changes after that. Here, we examined the prevalence of PNN-surrounded neurons in particular areas of animals of different ages. Our sample size is limited so we cannot perform a statistical analysis but, as Figure 11 shows, it is apparent that for nearly all areas there is no large change in PNN prevalence with age. Though the differences that we saw with age might reflect only between-animal variability, large monotonic changes seem likely to show a real change. One area in which we see such differences is in the deep layers of the superior colliculus. The percentage of neurons in the superior colliculus increases monotonically from age 5 to 20 and is over four times greater at age 20 than at age 5. Also, both the cingulate cortex and area MT appear to lose PNNs with age.

**Figure 12:**
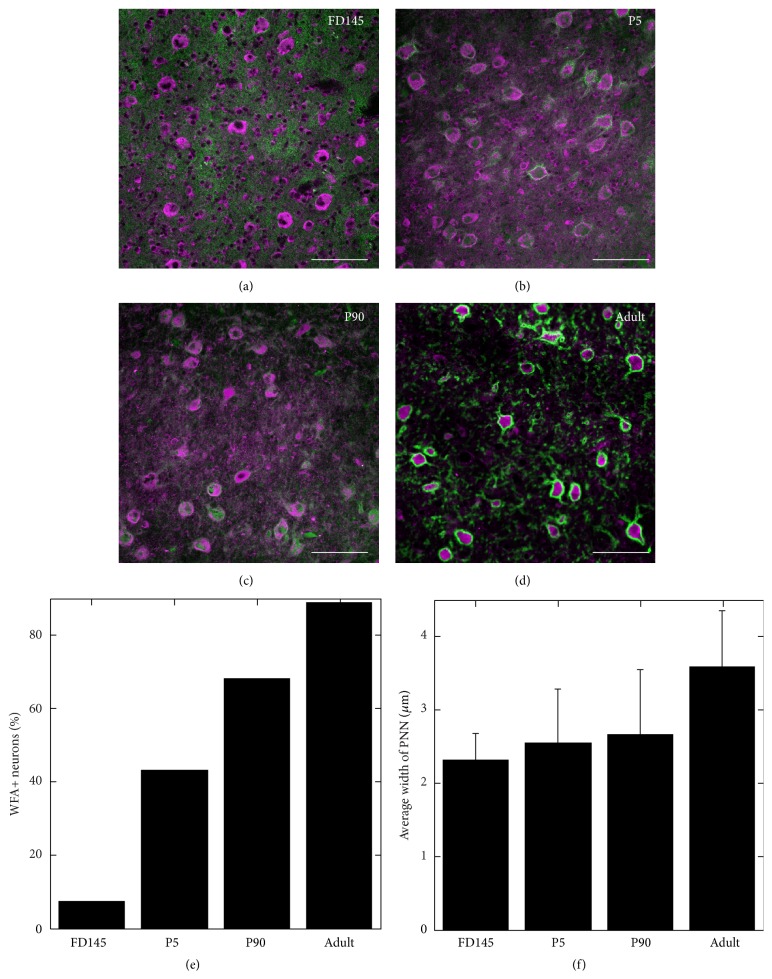
Perineuronal nets in the developing cerebellar nucleus. Perineuronal nets (stained with WFA, green) accrue around neurons in the cerebellar nuclei (stained with avidin, pink) between (a) fetal day 145, (b) postnatal day 5, (c) postnatal day 90, and (d) the adult animal. (e) shows the number of neurons surrounded by nets in (a)–(d). (f) shows the width of PNNs around cells in (a)–(d). Scale bars in (a)–(d) = 100 *μ*m.

We examined the formation of PNNs around neurons in the cerebellar nuclei during development. Figures 12(a)–12(d) show superimposed WFA (PNN) and avidin (neuron) labeling in the cerebellar nuclei at four ages between fetal day 145 and adult. Birth is at about day 164.  Figure 12(e) shows that the percentage of neurons surrounded by nets increases rapidly in the 24 days between FD145 and P5 and changes little after that. Our WFA staining shows earlier formation of PNNs than does staining with CAT-301 which found that CAT-301+ nets do not form in monkey cerebellar nuclei until after P5 [16]. We believe that this difference occurs because WFA stains components of PNNs that develop earlier than those stained by CAT-301. When we costained tissue from monkeys at different ages with both CAT-301 and WFA, we found that WFA marked PNNs earlier than did CAT-301.

In contrast, as Figure 12(f) shows, the width of PNNs increases between postnatal day 5 and the adult. Nets are significantly wider in age P90 tissue than P5 (*p* < 0.01), but not significantly different between FD145 and P5 sections (*p* = 0.46). There is also a significant difference (*p* = 0.02) between P90 and adult PNN widths, with the adult being larger. We collected these data from only one animal each. We therefore cannot provide any information about the variability in the developmental timeline between animals.

## 4. Discussion

### 4.1. Technical Considerations

Although we used an atypical neuronal marker to identify neurons in the cerebellar nuclei (based on McKay et al. [51]), we used this marker exclusively for quantification in this region, an area in which endogenous biotin expression is exceptionally high. Although avidin, which recognizes biotin, is not a standard neuronal stain, costaining with NeuN in regions outside the cerebellum indicates that avidin labels the same number of neurons, or slightly more so than does NeuN. Thus, if anything, our use of this stain may generate a slightly higher estimate of the number of neurons present than does NeuN.

In extremely rare instances, we appeared to find WFA staining that did not encapsulate a neuron. There are several possible explanations. First, a difference in the ideal focal plane during image capture would result in good signal of the extracellular PNNs, but potentially a very weak signal of the spatially displaced neuronal nuclei marker. Second, it is possible for PNNs ensheathing processes to form a circle that looks like a cell but would not express a neuronal marker. Third, although it has never before been established, it is possible that PNNs also surround cells that do not express typical neuronal markers. Regardless of which of these cases is correct, for this study, we counted neither the (absent) cell nor the PNN it putatively ensheathed.

### 4.2. Perineuronal Nets Are More Prevalent in Motor Structures

Among the areas that we examined, PNNs generally surrounded a larger proportion of neurons in motor areas than in sensory areas. M1 neurons have more nets than S1, A1, or V1. This was especially noticeable in the spinal cord, where we saw a greater-than-40-fold difference in the proportion of neurons surrounded by PNNs across a distance of less than a millimeter. Although our results appear to differ from those of Alpár et al. [56], who found a higher density of PNNs in rat primary visual and somatosensory cortex than elsewhere in the brain, our different methodologies explain this. Alpár et al. calculated density by counting cells with nets per 1 mm^2^, whereas we counted the fraction of neurons with nets in a given area. Since there are far more neurons per mm^2^ in primary visual cortex than in other areas of the brain [57], this could easily explain our apparently different estimates.

In addition to more PNNs in motor than sensory structures, we found that the highest proportion of neurons surrounded by PNNs was in the cerebellar nuclei, a motor structure. If we assume that the function of PNNs in these structures is to inhibit plasticity, then it is possible that sensory input is more changeable than motor output and that it is beneficial to have fewer nets and more plasticity present in sensory areas. Those areas might require more flexibility in synaptic connectivity than motor areas.

### 4.3. Implications of Differential Perineuronal Net Expression for Plasticity

PNNs are thought to play a role in restricting plasticity [58–61]. They prevent new nerve fibers and cones from connecting with the postsynaptic cell [62, 63] (reviewed by Rhodes and Fawcett [64]). Further, removal of PNNs allows lateral diffusion of AMPA receptors [65] and allows sprouting in the spinal cord [66, 67]. They mature around synapses during critical periods of development [6, 14]. Supporting this view is the observation that dissolving PNNs with the enzyme ChABC (chondroitinase ABC) causes sprouting and allows restructuring of connections that is similar to what occurs during critical periods in development [2, 3, 7, 68].

One likely candidate for a receptor through which PNNs could mediate inhibition of synaptic plasticity is PTP*σ*. Shen et al. [21] showed that the transmembrane protein tyrosine phosphatase PTP*σ* binds CSPGs. Further, cultured neurons without the PTP*σ* gene exhibited reduced inhibition (quantified by neurite outgrowth) by PNNs. Disruption of the PTP*σ* gene after spinal cord injury enhanced the ability of axons to penetrate CSPG-rich regions. Also, Liu et al. [34] found that visual deprivation during development resulted in a delay in the end of the visual critical period and PNN maturation, which coincided with stalling of PTP*σ* expression at critical period levels.

We consider the cerebellum a motor structure because it strongly influences movement via projections to premotor networks in the brainstem and to the origins of the four major descending motor tracts, the corticospinal (via relay in the ventral lateral nucleus of the thalamus), rubrospinal, vestibulospinal, and reticulospinal tracts. The cerebellum is also strongly implicated in motor adaptation. For example, during adaptation of saccade size, neurons in the saccade-related part of the cerebellar nuclei change their output in a way that would cause the observed change in saccade size [69]. This altered output almost certainly affects saccade size and is not just correlated with the change. Inhibitory burst neurons relay these changes to the motoneurons for the lateral rectus muscle in the abducens nucleus [70]. Blocking saccade-related cerebellar output blocks saccade adaptation [71]. Thus, the cerebellar nuclei represent a paradox. They are the structures with the highest percentage of neurons surrounded by PNNs but may also participate in motor adaptation that requires plasticity.

Two pieces of evidence suggest that PNNs in the cerebellar nuclei serve a purpose other than inhibiting synaptic plasticity. (1) We recently showed that dissolving PNNs in the cerebellar nuclei had no impact on the strength or persistence of changes in saccade size elicited by long term saccade adaptation [72]. (2) As we show in Figure 10, one of the ligands most likely to mediate inhibition of synaptic plasticity, PTP*σ*, is not expressed in the cerebellar nuclei in a manner consistent with this function. Therefore, at least in the cerebellar nuclei, PNNs seem to serve a function other than inhibition of synaptic plasticity.

### 4.4. Implications of Differential Perineuronal Net Expression for Neuroprotection

PNNs may also serve a neuroprotective role. Cabungcal et al. [22] used mice carrying genetic imbalance to demonstrate that PNNs around parvalbumin+ interneurons play a critical role in protecting these neurons from oxidative stress. PNNs limit the effect of genetically impaired antioxidant systems and/or excessive reactive oxygen species in the cell's environment. Parv+ cells without nets are more susceptible to oxidative stress.

Some evidence showed that components of PNNs can interact with iron, which is involved in the generation of reactive hydrogen radicals [23, 73, 74]. Also, PNN CSPGs act as a ligand for extracellular superoxide dismutase (EC-SOD) [24, 25]. EC-SOD catalyzes the dismutation of superoxide radicals which would otherwise damage proteins in the extracellular matrix and plasma membrane.

Another possibility is that PNNs act as a cation sink, surrounding neurons that either sustain or achieve very high firing rates, such as parvalbumin+ interneurons. They could therefore act as a buffering system for the rapid cation exchanges that occur in the extracellular space local to highly active neurons [26, 35]. Consistent with this idea are the many studies that show that PNNs surround fast-spiking parvalbumin-positive interneurons [13–15, 22, 32, 33]. Although our data is consistent with these results for some areas, we also show that for many areas the presence of parvalbumin in a cell does not correlate with PNN presence. Also, we did find that neurons in the inferior olive, small neurons that fire at very low rates (approximately 1-2 Hz), do not have PNNs, while neurons in the cerebellar nuclei, large neurons that fire tonically at rates of 60–100 Hz, did. Purkinje cells in the cerebellar cortex, which have similarly high tonic firing rates, are not surrounded by PNNs at all. This demonstrates there is no simple relationship between the metabolic properties of a cell and its likelihood of being surrounded by PNNs.

### 4.5. Implications of Differential Perineuronal Net Expression for Ion Homeostasis

Recent data point to a third possible PNN function. The impermeant anions of the cytoplasm and the strongly anionic extracellular PNNs contribute to setting the Cl^−^ reversal potential in neurons [43]. They therefore also determine the polarity of response to GABAergic input to the cell, which shifts during development. Glykys et al. [43] found a negative correlation between the “intensity” of PNN presence and the internal Cl^−^ concentration, an expected result from PNNs setting the local extracellular Cl^−^ concentration. They also found that digesting PNNs increased the internal Cl^−^ concentration more than threefold. The reduction in the internal Cl^−^ concentration during development parallels the increase in neuronal cytoplasmic anions during development [75] and experience [76] and increases in PNNs [65]. Further, there is the correlation between the ending of the critical period and the shift in the polarity of GABA [77, 78]. Also consistent with this possibility is the finding by Dityatev et al. [13] that degradation of PNNs resulted in an increase in the excitability of interneurons.

PNNs may surround neurons for which it is important that the Cl^−^ potential be maintained. This would be particularly important for neurons which receive substantial inhibitory input, which results in fluctuations of Cl^−^ across the membrane. Thus, PNNs around GABAergic synapses would ensure that these connections remain inhibitory. Our data support such a possibility. In some areas of the brain, the majority of synapses on parvalbumin+ neurons are GABAergic [79], as are the synapses on cerebellar nuclear neurons [16, 28]. However, we also found that neurons in the inferior olive are not surrounded by PNNs. Previous work [80] shows that inferior olive cells receive substantial GABAergic input from neurons in the contralateral cerebellar nuclei. We cannot therefore conclude that there is a simple relationship between the amount of inhibitory input a cell receives and the degree to which it is surrounded by PNNs.

### 4.6. Comparison of PNN Distributions in Macaques and Other Species

Although this is the first survey of PNN expression across the entire primate brain, several other studies have examined the expression of PNNs in isolated areas and in other species. Unfortunately, many of these reports do not provide cell counts but register only absence or presence of PNNs in a given area.

Many previous studies find PNN expression in the rodent cerebral cortex [81], especially in layers 2–4 [82, 83]. Also, although they do not give specifics regarding the subregions they examined, McGuire et al. [84] found between 3.02 and 14.44% of cells labeled with PNNs in posterior parietal areas. This is similar to our finding that 3–10% of neurons in various regions of the monkey cerebral cortex are surrounded by PNNs. Bertolotto et al. [83] quantified cells/mm^2^ for motor, somatosensory, and visual cortex. These numbers in the rodent are very different from those in the monkey and it is not possible to directly compare our results to theirs. Like Bertolotto et al., we found that the overall density (surrounded neurons/mm^2^) of PNN+ cells/area was highest in the V1. Depending on which layers of motor and somatosensory cortex we examined, we could find higher densities of PNNs within either motor or somatosensory cortex.

Although we examined only two regions of the nonhuman primate frontal cortex, the FEF and the orbital gyrus, McGuire et al. [84] performed an exhaustive examination of aggrecan expression in the frontal cortex of macaques using the Cat-301 antibody. They classified aggrecan staining in each cortical area as falling into one of two categories, cellular labeling in layers 3 and 5 or diffuse labeling in layers 2–5. These two categories also differed in the relative numbers of pyramidal to nonpyramidal cell labeling. The FEF, primary motor cortex, SMA, and premotor cortex were all in the first profile while the orbital gyrus, dlPFC, and cingulate cortex were in the second. They found the most intense staining in the PFC frontal cortex in FEF, which was also our finding. McGuire et al. used different counting procedures so it is not possible to directly compare their counts with ours. Still, it appears that they often found higher prevalence of PNNs than we did. This could be due to either their experimental procedure or our having different criteria for what qualifies as a labeled cell. Further, Cruz-Rizzolo et al. [85] performed a detailed qualitative examination of perineuronal net expression in the monkey* Cebus apella* and found less staining in orbitofrontal cortex than putative frontal eye field, as well as highly diverse expression of PNNs throughout the frontal cortex. Also, although Seeger et al. [86] appeared to find similar levels of WFA+ cells throughout the rat cortex, Brückner et al.'s findings [87] in human are again more similar to our own in that far fewer PNNs were found in frontal cortex and association areas than in primary sensory and motor areas.

Vitellaro-Zuccarello et al. [88] and Seeger et al. [86] found that the distribution of PNNs throughout the thalamus was very variable. This does not conflict with our results. We examined only a small subset of the thalamic regions, medial dorsal and ventral posterolateral, but Brückner et al. [89] found WFA staining in the reticular thalamic nuclei in mouse, but less conclusive results using other PNN markers. According to Gáti et al. [90], who examined PNN expression in rat thalamus, PNNs are often absent from traditionally defined relay regions of the thalamus and more prevalent in regions that connect directly to primary cortical regions. Our findings also qualitatively match those found by Seeger et al. [86] (rat), Adams et al. [91] (macaque), and Brückner et al. [92] (human), who examined perineuronal net presence in the primate basal ganglia.

Bertolotto et al. [83], like us, found very little evidence of PNN presence in the hippocampus. However, Brückner et al.'s [89] study in mouse found staining in CA1–3, as did Seeger et al.'s [86] study in rat. This does not conflict with our result because our counts were restricted to the dentate gyrus. Indeed, Lendvai et al. [93] found that, qualitatively, the dentate gyrus has far fewer PNNs than CA1–CA3, the subiculum and entorhinal cortex in human tissue. Ajmo et al. [82] also examined PNN staining in the mouse and rat hippocampus and like us found only scattered neurons labeled with WFA.

Several reports are also in agreement with us regarding the expression of PNNs in the adult cerebellar cortex. Popp et al. [81], Aquino et al. [94], Seeger et al. [86], and Bertolotto et al. [83] all found low or no expression of key PNN proteins around cerebellar Purkinje cells. Like us, Bertolotto et al. found a high density of staining around neurons in the cerebellar nucleus of the mouse. Also, like us, Brückner et al. [89] and Seeger et al. [86] identified strong labeling of PNNs with WFA in many regions of the brainstem including the cerebellar and vestibular nuclei, the colliculi, and the pons.

Galtrey et al. [7] examined the expression of PNN proteins in the rat spinal cord using WFA and NeuN, as we did. They found that PNNs surrounded fewer motoneurons compared to what we found (30% versus 76%) and that a large number of interneurons in the intermediate grey were also surrounded (50%, unquantified in our case) and 20% of neurons in the dorsal horn were surrounded. We found almost no evidence for PNN expression in the dorsal horn, but it is possible that this is a result of our differing demarcations of this zone. Galtrey et al. also noted that there were no PNNs in the cord's dorsalmost laminae. Also, like us, they found diffuse WFA staining around neurons with the tight WFA-labeled ensheathments. This might have made identifying labeled neurons more difficult. Similarly, when Jäger et al. [95] examined PNNs in the human spinal cord, they also found more aggrecan ventrally than dorsally and, as we did, found that not all ChAT+ neurons were also PNN+ (71% in their case, 76% in ours).

To date, very few studies have examined PNN presence in humans. Bertolotto et al. [83] compared their findings in rat to human sample and found very similar results, with the exception that they also identified labeling around some large pyramidal neurons in layer V of the cerebral cortex, as we do in the rhesus macaque, and note that this is something they did not witness in rat tissue.

Our results qualitatively match those of other studies examining PNN presence in different parts of the central nervous system in rodents and humans, with the exception of the inferior olive. Where our results differ, it is likely that this is the result of either interspecies variation or differences in techniques between labs, that is, the use of other markers for PNNs. Overall, this suggests that PNN prevalence is broadly maintained across taxa.

### 4.7. PNNs around a Small Percentage of Neurons Inhibit Plasticity

Finally, two previous studies restore critical period-like plasticity in V1 [2, 68] and the amygdala [3] of adult rats by dissolving PNNs in these areas. Dissolving PNNs in V1 allowed visual experience to change in the distribution of visual input to V1 and to improve the acuity in a rat's occluded eye [2]. Dissolving them in the amygdala allowed experience to erase fear conditioning in adult rats, conditioning previously thought to be permanent [3]. These striking changes in behavior and brain structure occur after removal of PNNs from brain areas in which, in macaque, only 5% (V1) and 3% (amygdala) of neurons are surrounded by PNNs.

These changes were experimentally induced in rats and it is possible that PNNs surround a much higher percentage of neurons in V1 and amygdala in rat than they do in macaque. We assessed the proportion of PNNs in V1 and amygdala in one rat using the same methods that we used for macaques. We found that in this animal PNNs surrounded 3.5% of V1 neurons and <1% of amygdala neurons.

The fact that WFA+ PNNs surround only a small proportion of neurons in V1 and the amygdala may mean that the large increases in plasticity demonstrated after injecting chondroitinase result from dissolving the PNNs around only this small proportion of neurons. Alternatively, the injected chondroitinase may dissolve the chondroitin sulfate chains in both the PNNs and the rest of the extracellular matrix within the injection site. It is possible that destruction of this less salient but more broadly distributed matrix, not just the PNNs, increases plasticity. In the absence of more evidence about the function of PNNs, it is just as plausible that the widespread extracellular matrix helps inhibit plasticity as it is that PNNs around only 5% of neurons inhibit plasticity. It will be worthwhile to rule out the former possibility.

## 5. Conclusion

In summary, the density of PNN-ensheathed neurons is very different in different areas of the central nervous system. No generalization proposed so far accounts well for these differences because we do not yet understand the specialized functions of neurons in these regions, or of PNNs. It appears that in some areas PNN presence around only a small proportion of the neurons is sufficient to block plasticity. However, in other areas, such as the cerebellar nuclei, PNN presence is probably not related to plasticity, but maybe instead to local ion homeostasis.

Because no single explanation can currently account for PNN presence around neurons throughout the central nervous system, it is likely that PNNs serve different functions in different regions of the brain.

## Supplementary Material

The supplementary material shows PNN expression in other areas of the macaque brain and, for a subset of regions, its localization with respect to Parvalbumin+ neurons. It also contains two figures that demonstrate the reliability of our staining technique.

## Figures and Tables

**Table 1:** Percentages of WFA+ neurons by region and animal. The top rows detail each animal's sex and age. The subsequent rows show the percentage of neurons that were surrounded by WFA+ PNNs for each region in each animal that we collected data from.

	Animal								
	A	B	C	D	E	F	G	H	I
Sex	Male	Male	Male	Female	Male	Male	Male	Male	Male
Age	9	8	5	12	5	7	6	21	11

*Region*									
Primary auditory cortex (A1)	3.91		3.62		3.54		4.56		
Primary motor cortex (M1)			13.12	8.20	8.70	9.14			
Primary somatosensory cortex (S1)	7.16	6.14	6.21	6.66	5.93	11.72			
Primary visual cortex (V1)	4.66	4.81	6.19	6.14	5.36	5.53			
Frontal eye field (FEF)	8.03	2.96	8.88	5.52	6.44	3.49			
Cingulate cortex	2.24		5.18	0.80		5.72			
Mediotemporal cortex (MT)		4.50	5.65	3.26	7.65	4.43			
Orbital gyrus	5.02	2.75	7.72	5.71	5.94	5.73			
Amygdala	5.26	2.88	0.25	0.81	3.86				
External globus pallidus	8.20		18.76		12.84		62.22	12.86	
Internal globus pallidus			14.95		86.49		100.00	67.20	
Hippocampus	1.06		2.92		2.82	0.88			
Thalamus	1.66	0.73	0.00	4.05	0.00	0.00			
Putamen	2.57		0.00		1.22		1.13	0.00	
Inf. colliculus (deep)		6.63	7.41					8.40	10.30
Inf. colliculus (sup.)		5.84	0.67					3.81	5.89
Sup. colliculus (deep)		6.91	2.52					19.91	15.63
Sup. colliculus (sup.)		0.74	6.38					2.60	3.41
Inferior olive (IO)	0.00	0.00	0.00						0.00
Cerebellar nucleus	95.60	91.67	98.08	88.03					
Vestibular nucleus	61.73	62.58	40.00					66.67	40.23
Pontine nuclei	5.47		28.37		43.58			42.25	25.59
Ventral spinal cord	36.60	42.39	43.71	44.62	41.78	55.63			
Dorsal spinal cord	0.19	1.46	1.57	0.51	3.42	1.74			
