# Supplementary material for: Distribution of N-Acetylgalactosamine-Positive Perineuronal Nets in the Macaque Brain: Anatomy and Implications
Source: Neural Plast. 2016 Jan 3;2016:6021428. doi: 10.1155/2016/6021428 (PMC4735937; doi:10.1155/2016/6021428)

Supplementary Figure Legends

Figure S1: WFA and Aggrecan (Cat-301) antibody co-stain cells in the cerebellar nuclei and ventral spinal cord. The top panels show costaining of neurons in the cerebellar nuclei. The bottom panels show costaining of neurons in the ventral spinal cord. Scale bars = 100µm.

Figure S2: Perineuronal nets in cortex. The left panels show perineuronal nets stained with WFA. The middle panels show anti-NeuN-labeled neurons. The right panels show merged images of the two. Abbreviations: cingulate cortex (Cing. Ctx), frontal eye field (FEF), medio-temporal cortex (MT) and cortex from the orbital gyrus (Orb. Gyrus). Scale bar = 200µm.

Figure S3: Perineuronal nets in subcortical areas. The left panels show perineuronal nets stained with WFA. The middle panels show anti-NeuN-labeled neurons. The right panel shows merged images of the two. Scale bar = 200µm.

Figure S4: Perineuronal nets in the basal ganglia. The left panels show perineuronal nets stained with WFA. The middle panels show anti-NeuN-labeled neurons. The right panel shows merged images of the two. Abbreviations: external globus pallidus (ext. GP), internal globus pallidus (int. GP). Scale bar = 200µm.

Figure S5: Perineuronal nets in the brain stem. The left panels show perineuronal nets stained with WFA. The middle panels show anti-NeuN-labeled neurons. The right panel shows merged images of the two. Abbreviations: vestibular nuclei (Vestib. Nuc), inferior olive (Inf. Olive). Scale bar = 200µm.

Figure S6: Perineuronal nets in the colliculi. The left panels show perineuronal nets stained with WFA. The middle panels show anti-NeuN-labeled neurons. The right panel shows merged images of the two. The top two rows show superficial and deep layers of the superior colliculi (Sup. Coll.). The bottom two rows show superficial and deep layers of the inferior colliculi (Inf. Coll.). Scale bar = 200µm.

Figure S7: Perineuronal nets and parvalbumin-positive cells in cerebral cortex. Upper panels: primary visual cortex (V1). Lower panels: Frontal eye field (FEF). The left panels show perineuronal nets stained with WFA. The middle panels show anti-parvalbumin-labeled neurons. The right panels show merged images of the two. Scale bar = 200µm.

Figure S8: Perineuronal nets and parvalbumin-positive cells in the cerebellum. Upper panels: cerebellar cortex (CBL Cortex). Lower panels: cerebellar nucleus (CBL Nucleus). The left panels show perineuronal nets stained with WFA. The middle panels show anti-parvalbumin-labeled neurons. The right panels show merged images of the two. Large parvalbumin-positive Purkinje cell bodies are evident in the cerebellar cortex. Scale bar = 400µm.

Figure S9: Perineuronal nets and parvalbumin-positive cells in the spinal cord. Upper panels: dorsal horn of spinal cord. Lower panels: ventral horn of spinal cord. The left panels show perineuronal nets stained with WFA. The middle panels show anti-parvalbumin-labeled neurons. The right panels show merged images of the two. Scale bar = 200μm.

Figure S10: High magnification view of neurons surrounded by perineuronal nets in several areas of the CNS. A1 is primary auditory cortex, S1 is primary somatosensory cortex, V1 is primary visual cortex and M1 is primary motor cortex. Scale bar = 30μm.

Table 1: Percentages of WFA+ neurons by region and animal. The top rows detail each animal's sex and age. The subsequent rows show the percentage of neurons that were surrounded by WFA+ PNNs for each region in each animal that we collected data from.

Supplementary Figure 1:

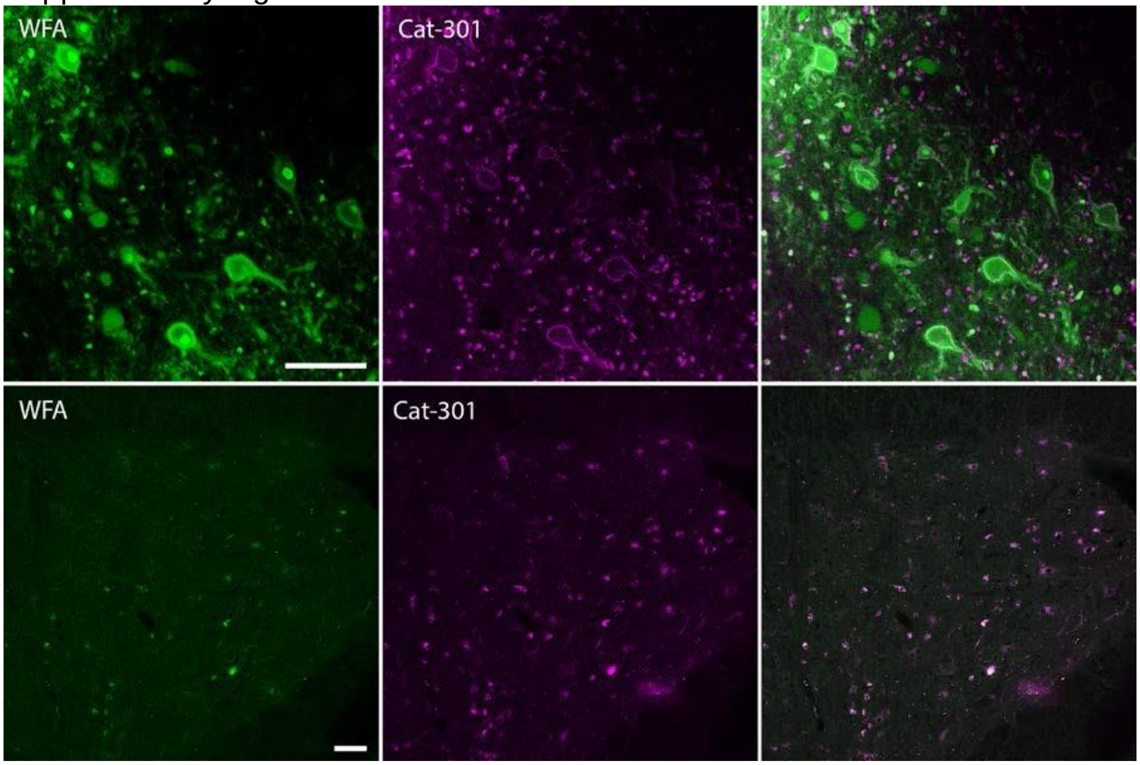

Supplementary Figure 2

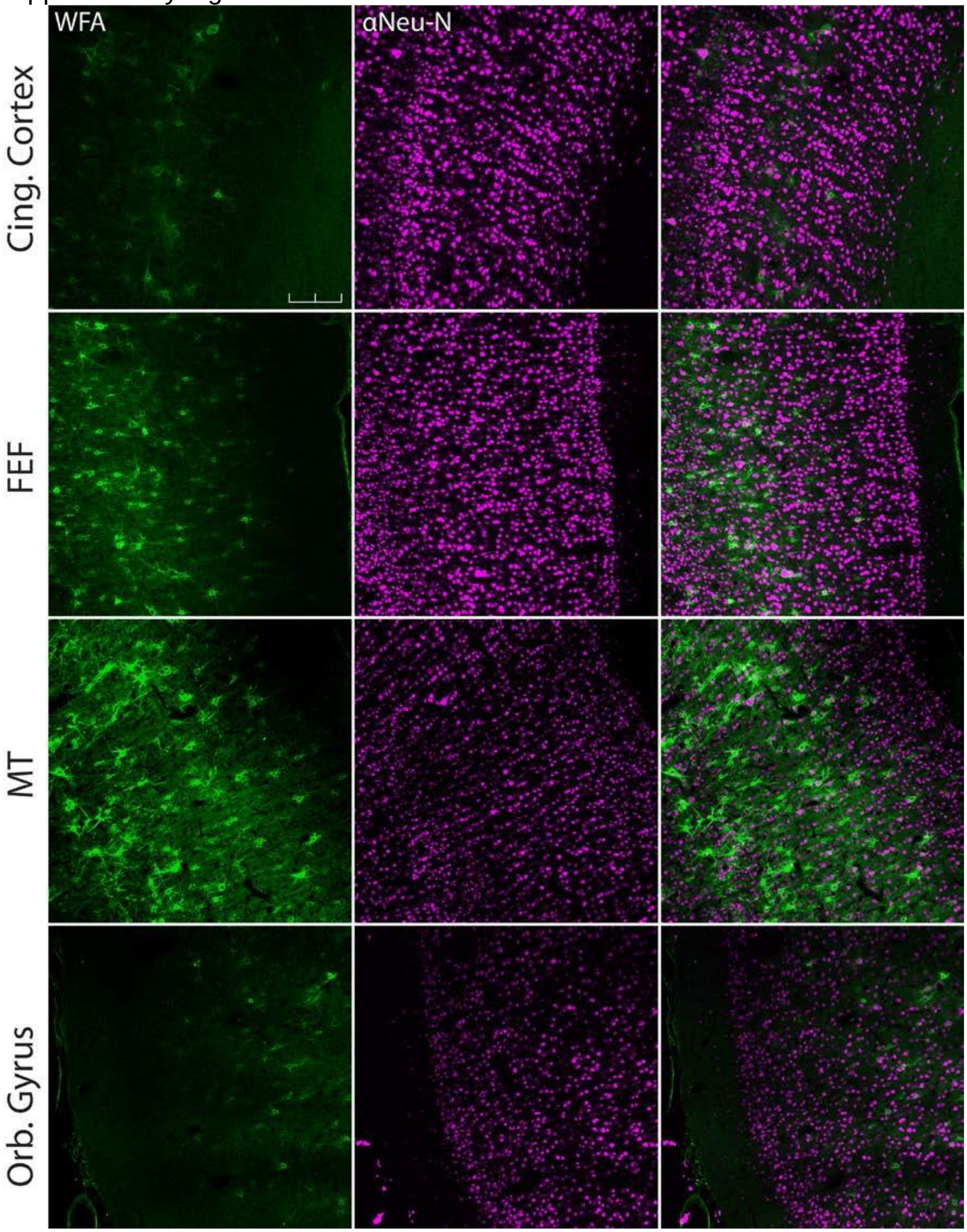

Supplementary Figure 3

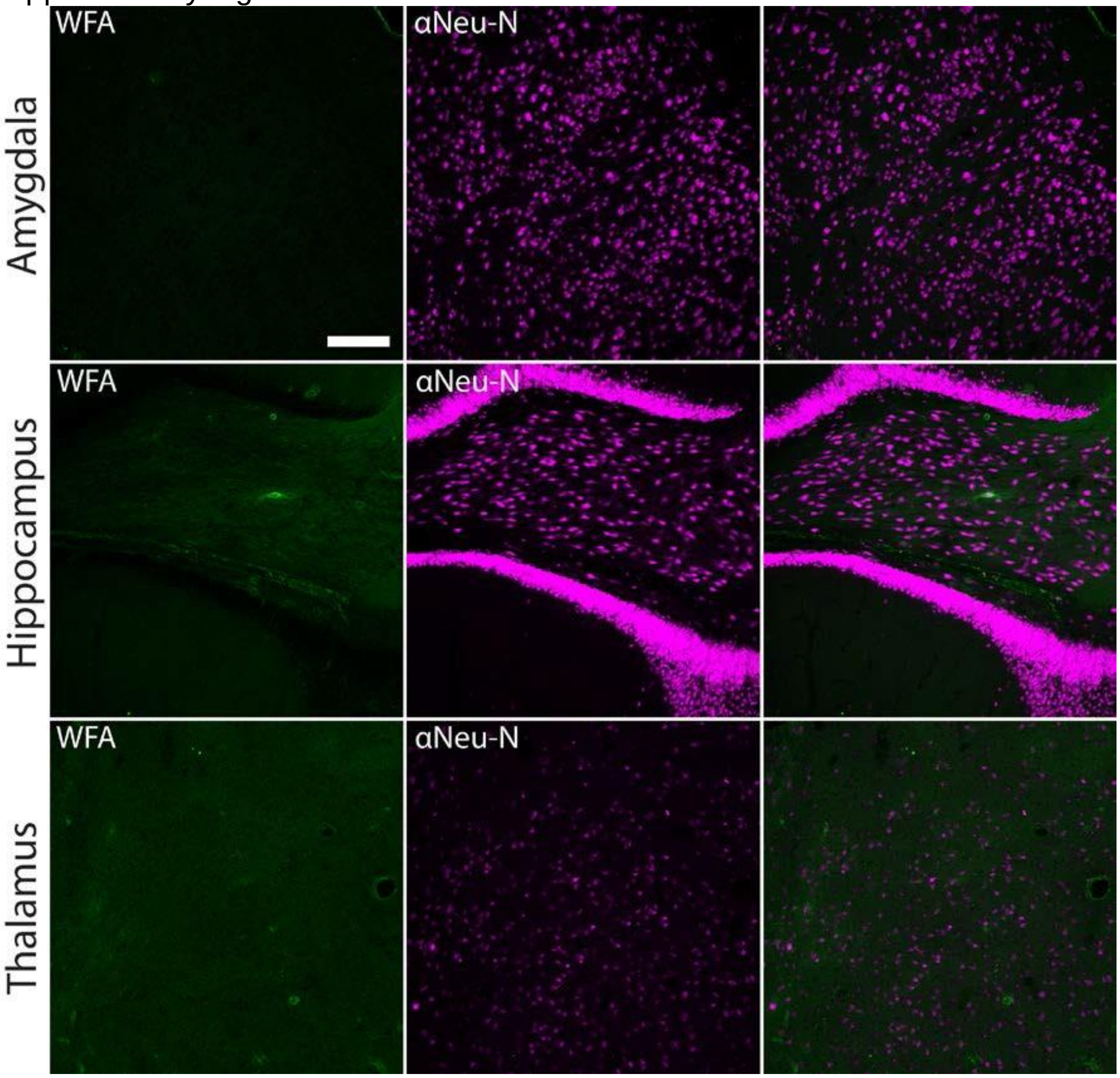

Supplementary Figure 4

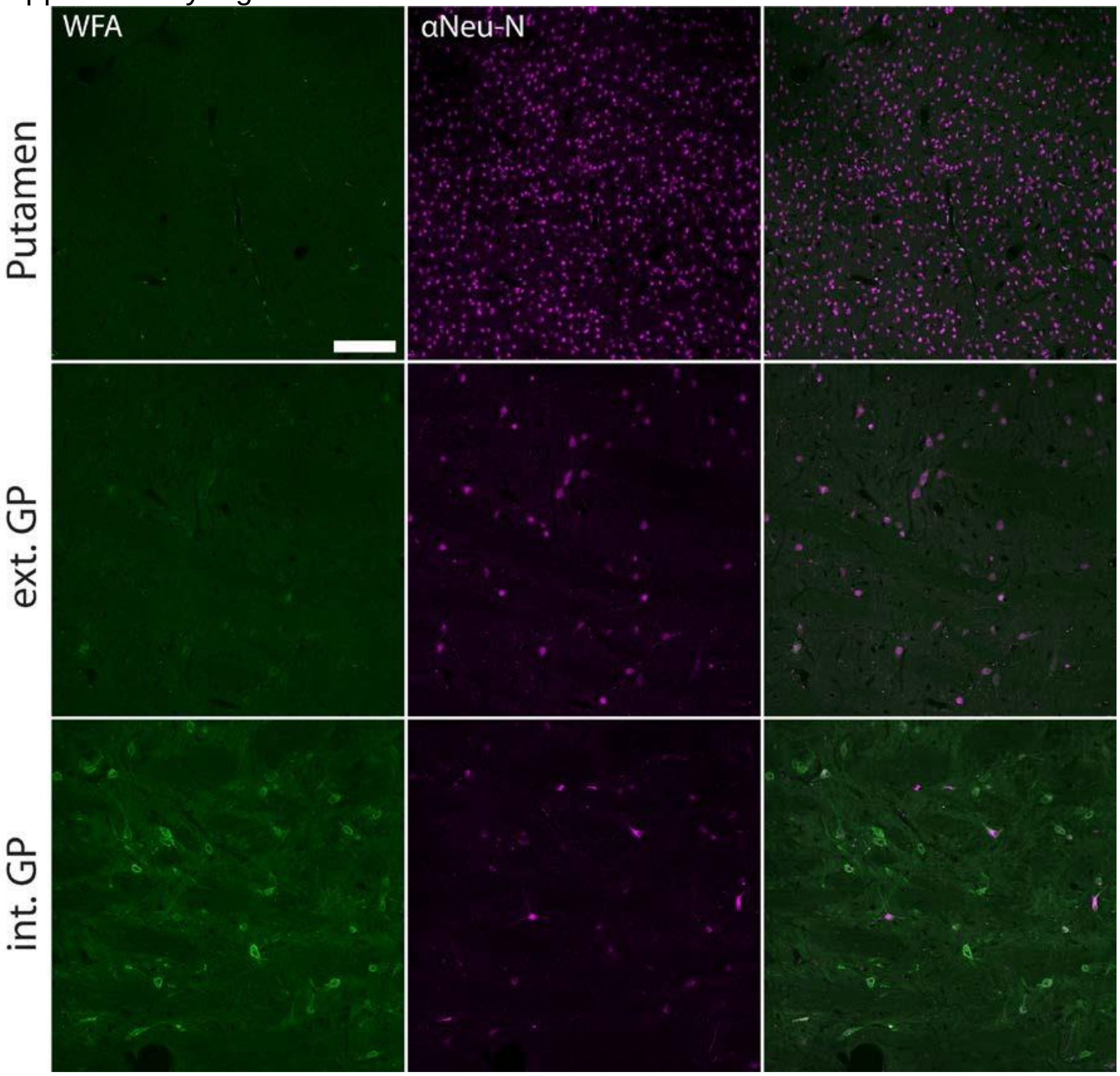

Supplementary Figure 5

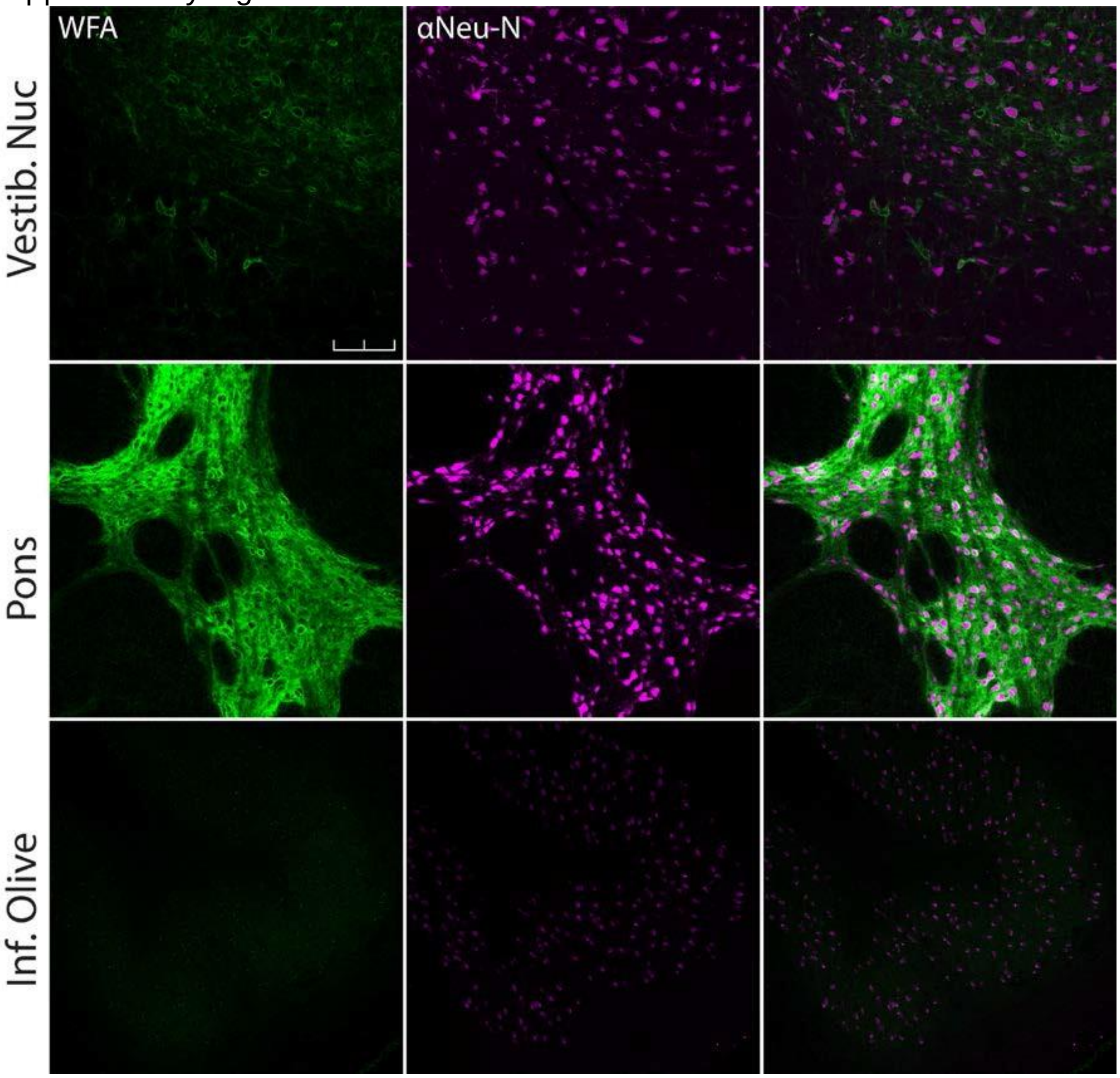

Supplementary Figure 6

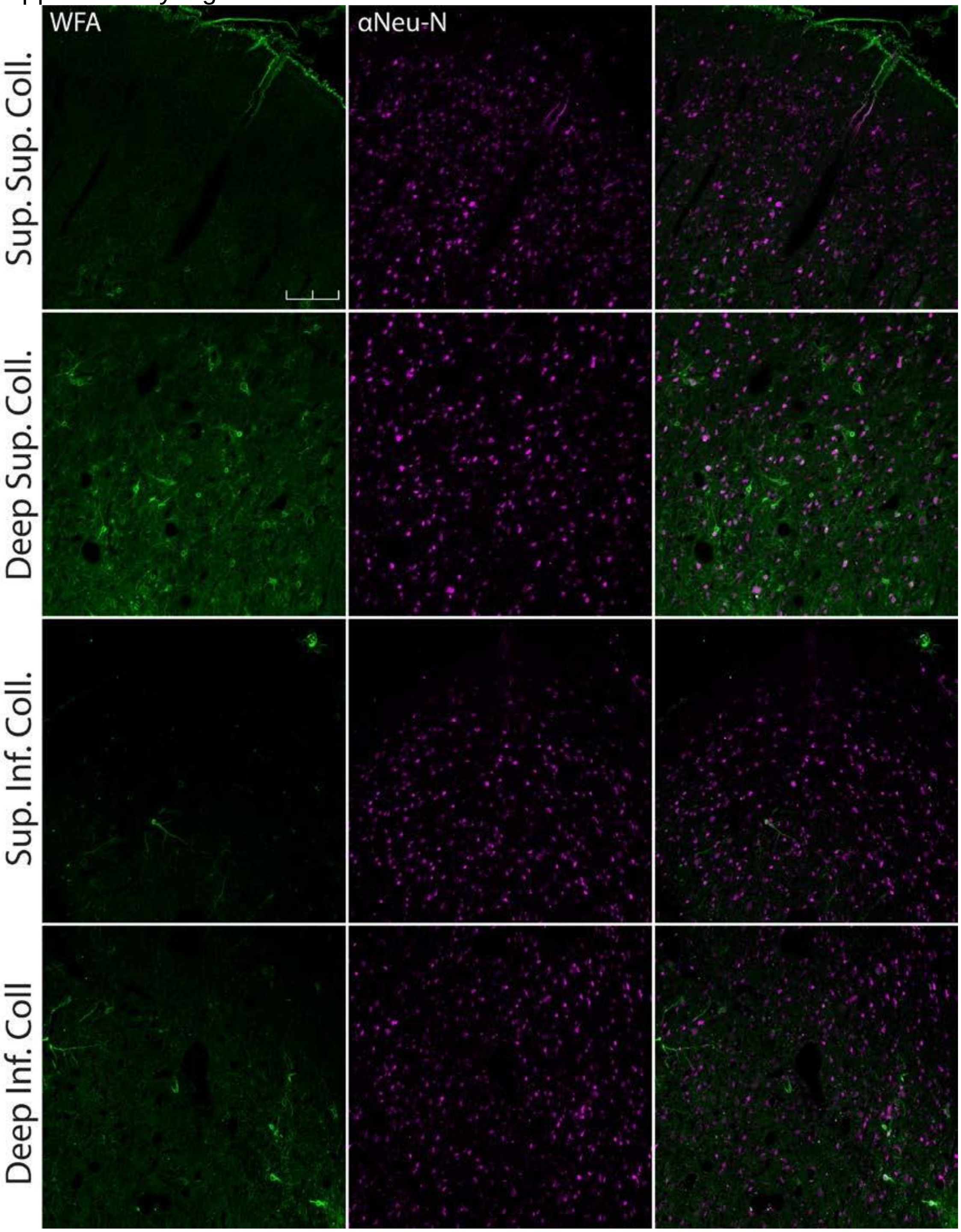

Supplementary Figure 7

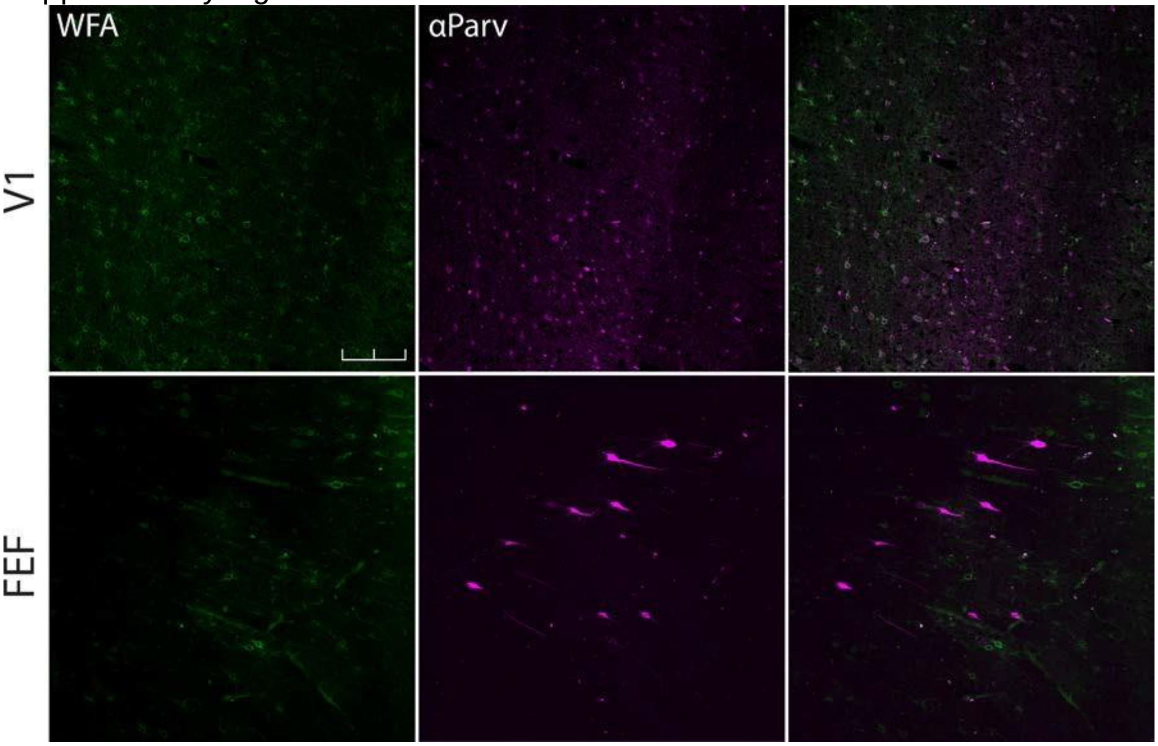

Supplementary Figure 8:

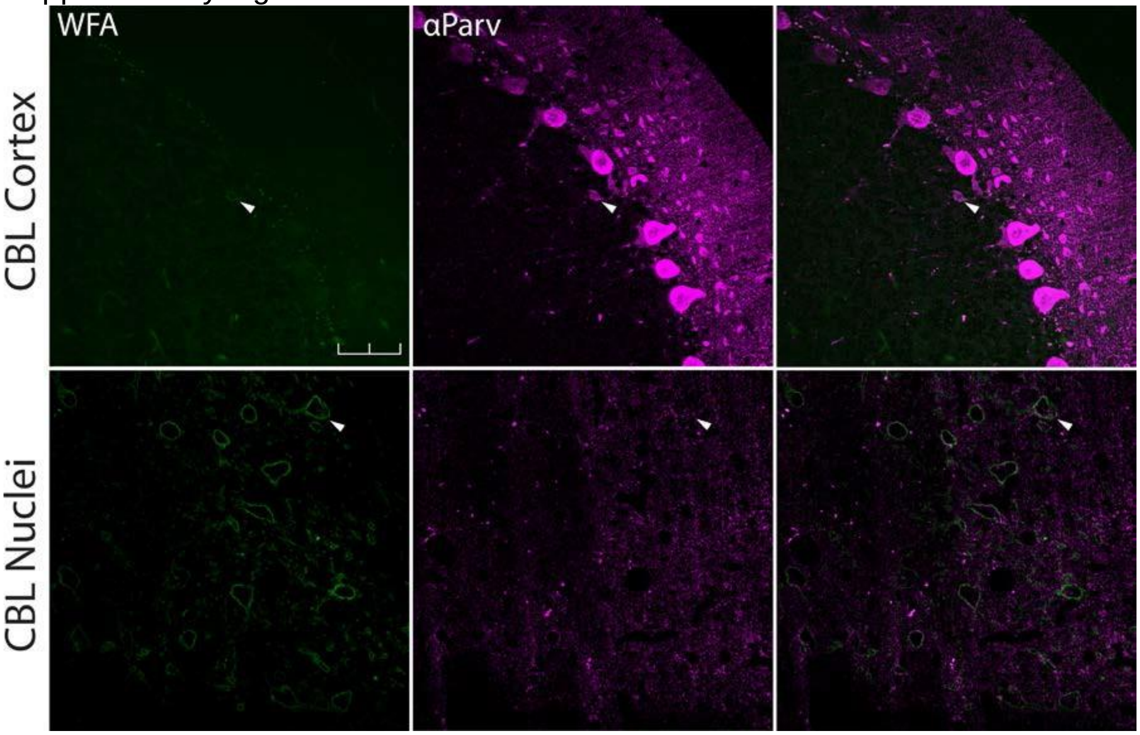

Supplementary Figure 9:

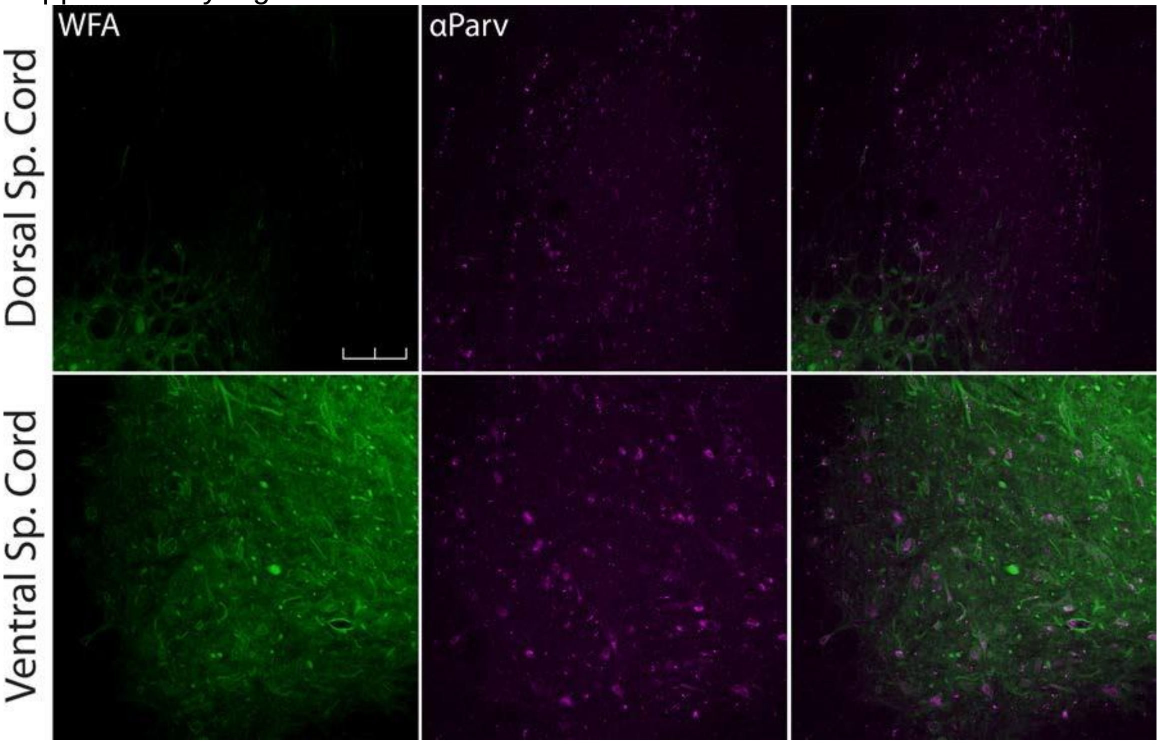

Supplementary Figure 10:

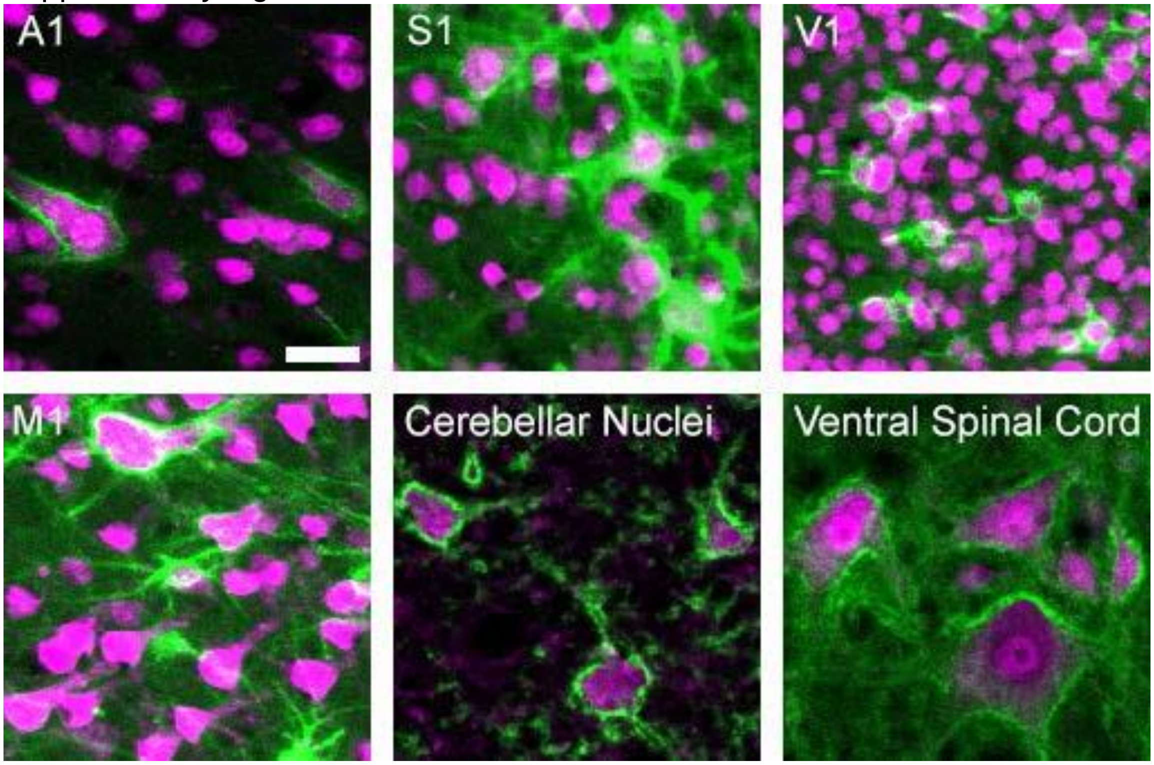

Supplement: Supplementary file 1 — The supplementary material shows PNN expression in other areas of the macaque brain and, for a subset of regions, its localization with respect to Parvalbumin+ neurons. It also contains two figures that demonstrate the reliability of our staining technique. [file 6021428.f1.pdf]
